# Novel Concepts for Drug Hypersensitivity Based on the Use of Long-Time Scale Molecular Dynamic Simulation

**DOI:** 10.1155/2016/9520361

**Published:** 2016-11-23

**Authors:** Takahiro Murai, Norihito Kawashita, Yu-Shi Tian, Tatsuya Takagi

**Affiliations:** ^1^Graduate School of Pharmaceutical Sciences, Osaka University, 1-6 Yamadaoka, Suita, Osaka 565-0871, Japan; ^2^Research Institute for Microbial Diseases, Osaka University, 3-1 Yamadaoka, Suita, Osaka 565-0871, Japan; ^3^Graduate School of Information Science and Technology, Osaka University, 1-5 Yamadaoka, Suita, Osaka 565-0871, Japan

## Abstract

The discovery that several drug hypersensitivity reactions (DHRs) are associated with specific human leukocyte antigen (HLA) alleles has attracted increasing research interest. However, the underlying mechanisms of these HLA-induced DHRs remain unclear, especially for drug-induced immediate activation of T-cell clones (TCCs). Recently, a novel hypothesis involving partial detachment between self-peptide(s) and the HLA molecule (altered peptide-HLA (pHLA) model) has been proposed to explain these phenomena. In order to clarify this hypothesis, we performed long-timescale molecular dynamics (MD) simulations. We focused on HLA-B⁎57:01-restricted abacavir hypersensitivity reactions (AHRs), one of the most famous DHRs. One of the simulation results showed that this altered-pHLA model might be driven by an increase in the distance not only between HLA and self-peptides but also between the *α*_1_ and *α*_2_ helices of HLA. Our findings provide novel insights into abacavir-induced immediate activation of TCCs and these findings might also be applied to other DHRs, such as HLA-B⁎58:01-restricted allopurinol hypersensitivity reactions.

## 1. Introduction

Administration of particular drugs sometimes causes drug hypersensitivity reactions (DHRs). Abacavir, a nucleoside reverse transcriptase inhibitor, plays an important role in anti-HIV regimens worldwide as one of the recommended antiretroviral drugs. However, due to its potential to cause DHRs, specifically named abacavir hypersensitivity reactions (AHRs), abacavir's safety profile has been questioned especially for use in children [1, 2]. A relationship between AHRs and certain alleles of human leukocyte antigen (HLA) has been reported. After administration of abacavir, AHRs occur in approximately 5 to 8% individuals carrying an HLA-B*∗*57:01 allele, which is a higher rate than the observed in people carrying other alleles [3–5].

Although many efforts have been made to investigate the mechanism by which AHRs occur, a complete explanation has not yet been obtained. According to previous studies, three distinct models have been reported: the hapten/prohapten model, the pharmacological interaction (p-i) model, and the altered repertoire model [6, 7]. Recent studies revealed that abacavir forms noncovalent bonds with HLA-B*∗*57:01. This binding may change the repertoire of the HLA-B*∗*57:01-binding peptides and trigger “foreign antigen recognition” by T-cells [8–10]; these findings supported the third model. However, none of the current three models can explain the fact that about 40 percent of AHRs derived from T-cell clones react within less than 15 minutes of abacavir treatment [11, 12]. All three current models suggest that a much longer reaction time should be necessary.

**Figure 1:**
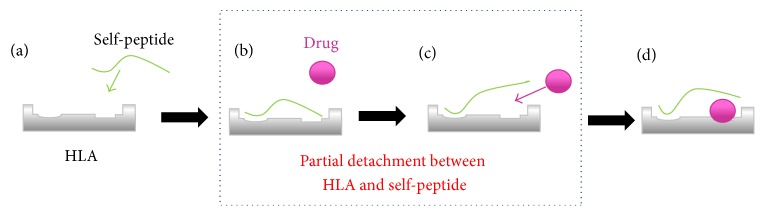
Schematic representation of the altered-pHLA model. First, self-peptides bind to HLA molecules in an HLA allele-dependent manner, forming the peptide-HLA complexes (a). Second, some peptide-HLA complexes undergo the partial detachments (b), enabling the accommodation of certain drugs into the space (c). Finally, peptide-drug-HLA complexes adopt stable conformations, leading to recognition by T-cells (d).

More recently, a hypothesis referred to as the altered peptide-HLA (pHLA) model has been proposed to explain this phenotype; a partial detachment between the HLA molecule and self-peptide(s) may exist [13]. This hypothesis implies that the drug binds not only to the HLA molecule in the endoplasmic reticulum (ER) of the antigen-presenting cell but also to pHLA complexes on the cell surface. The latter event could be a subsequent result of the dissociation between the peptide and the HLA, creating a pocket where the drug enters (Figure 1). The immediate activation of TCCs can be well explained by this hypothesis, because, instead of passing through the ER, a direct conformational change triggered by interaction of abacavir with pHLA could shorten the reaction time.

In order to clarify this hypothesis, we performed molecular dynamics (MD) simulations using the structure of the pHLA complex. One of the simulation results implied that certain self-peptides are partially dissociated from HLA-B*∗*57:01, enabling accommodation of abacavir and thereby stabilizing the peptide-binding cleft. Our findings provide novel insights into abacavir-induced immediate activation of TCCs.

## 2. Materials and Methods

### 2.1. Modeling of pHLA Complexes

The models of three self-peptides, which were shown to bind to HLA-B*∗*57:01 before and after abacavir treatment [8], were constructed using the LSSPVTKSF (LF9) peptide (Protein Data Bank (PDB) ID: 3VH8) as a template [14] (Table 1). Subsequently, the self-peptides were docked with the HLA-B*∗*57:01 (PDB ID: 3VH8) to model pHLA complexes* in situ*. During this docking procedure, the ASEDock program [15] was used; flexibility of the ligand atoms was allowed and the backbone atoms of the receptors were tethered. Following the docking scores (U_dock), the top-scoring pose of each docking was collected. A series of these models were performed using MOE 2013.08 software [16].

### 2.2. MD Simulations

MD simulations were performed using the GROMACS 5.0.4 software package 1 and the OPLS-AA force field [17]. The four structures of pHLA (including the crystal structure (PDB ID: 3VH8)) were soaked using the TIP3P water model. A dodecahedral box was selected with a minimum distance of 1.4 nm between the edges of the protein and the box, and each of the systems was neutralized by adding counter ions at physiological concentrations (0.15 M). The energy of each system was minimized by using the steepest descent algorithm for 100 ps. The* v*-rescale and Parrinello-Rahman methods were used to control temperature and pressure, respectively. The LINCS algorithm was used to constrain all bond lengths. The particle mesh Ewald (PME) method was used to compute long-range electrostatics. Finally, 500 ns MD simulations of all four complexes were performed at 310 K using the TSUBAME 2.5 supercomputer at Tokyo Institute of Technology [18].

## 3. Results

### 3.1. The Root Mean Square Deviation (RMSD) Values for Self-Peptides Complexed with HLA-B*∗*57:01 during MD Simulations

**Figure 2:**
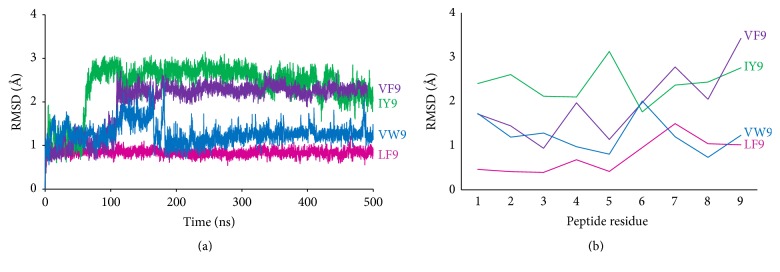
The RMSD values for the self-peptides. (a) RMSD values were calculated for C*α* atoms of the peptide during the MD simulations. (b) The RMSD values were calculated per residue for the C*α* atoms and averaged over 500 ns. Peptides VF9, IY9, VW9, and LF9 are shown in purple, green, blue, and magenta, respectively.

Conformational changes of the four investigated self-peptides bound to HLA-B*∗*57:01 were calculated by RMSD during the period of the MD simulations. For these peptides, VF9 and IY9 peptides showed larger conformational changes after 100 ns with peptide RMSD values of about 2 to 3 Å (Figure 2(a)). More detailed conformational changes of the four peptides were analyzed by calculating the RMSD per residue over a 500 ns simulation period (Figure 2(b)). For VF9 and IY9 peptides, the C-terminal residues (residues 7–9), which are located in the abacavir binding site, showed larger conformational changes with RMSD values of about 2 to 3 Å.

### 3.2. The Distance between the Specific Residues of Self-Peptides and Those of HLA-B*∗*57:01

**Figure 3:**
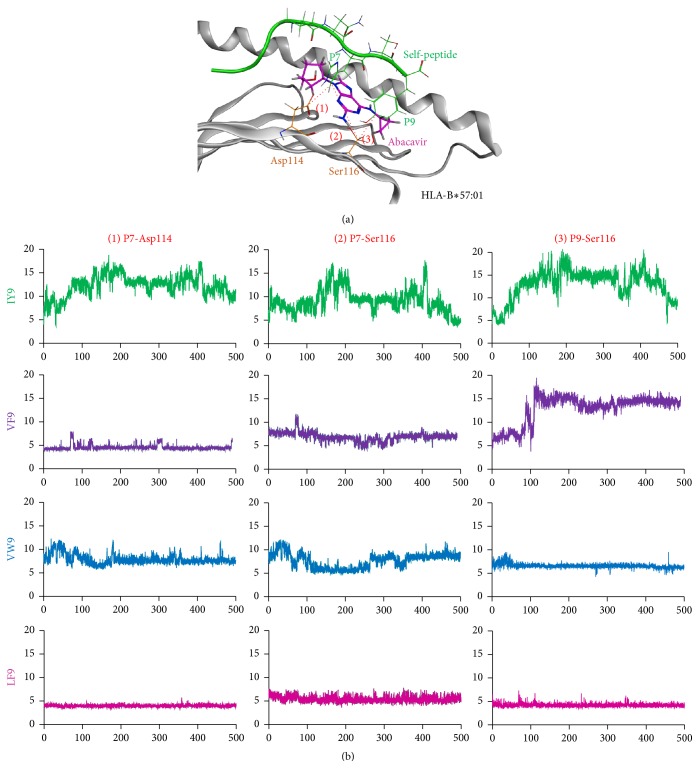
The distances between self-peptides and HLA-B*∗*57:01. (a) Measurements of the distances between the HLA molecule and self-peptides. The distances between the specific residues of self-peptides and those of HLA-B*∗*57:01 were measured during the MD simulations: (1) P7-Asp114, (2) P7-Ser116, and (3) P9-Ser116. HLA-B*∗*57:01 is shown in gray, and Asp114 and Ser 116, the HLA residues implicated in abacavir binding, are shown in orange. The self-peptide (IY9 peptide is shown here) and abacavir are shown in green and magenta, respectively. (b) The distance between self-peptides and HLA-B*∗*57:01. Horizontal and vertical axes of each graph represent time (ns) and RMSD values (Å), respectively. Color representation is the same as in (a).

In normal states, self-peptides bind to the peptide-binding groove of HLA-B*∗*57:01 and there is no enough space for a drug to bind. According to the novel hypothesis, some self-peptides dissociate from the HLA, enabling accommodation of certain drugs. To confirm the partial detachment between the HLA molecule and self-peptides, distances between the specific residues of self-peptides and those of HLA-B*∗*57:01 which are implicated in abacavir binding were calculated during the MD simulation. The residues examined were (1) P7- (peptide residue 7-) Asp114, (2) P7-Ser116, and (3) P9-Ser116 (Figure 3(a)). For these pHLA complexes, only the IY9 peptide-HLA-B*∗*57:01 complex resulted in larger distances compared with the initial distances (Figure 3(b)). The distances increased gradually after 100 ns and showed peak values around 200 ns (15.7 Å, 13.3 Å, and 18.1 Å, resp.), compared with their initial distances (4.0 Å, 6.8 Å, and 4.4 Å, resp.). Interestingly, the distance of P7-Ser116 after 200 ns (4.7 Å at 500 ns) and that of P9-Ser116 after 400 ns decreased (8.3 Å at 500 ns). For the VF9 peptide-HLA-B*∗*57:01 complex, while the distances of P9-Ser116 became greater after 100 ns, the others stayed constant during the MD simulation. These results imply that the IY9 peptide is likely to dissociate from HLA.

### 3.3. The Distance between HLA-B*∗*57:01 *α*_1_ and *α*_2_ Helices Complexed with the IY9 Peptide Increased during MD Simulation

**Figure 4:**
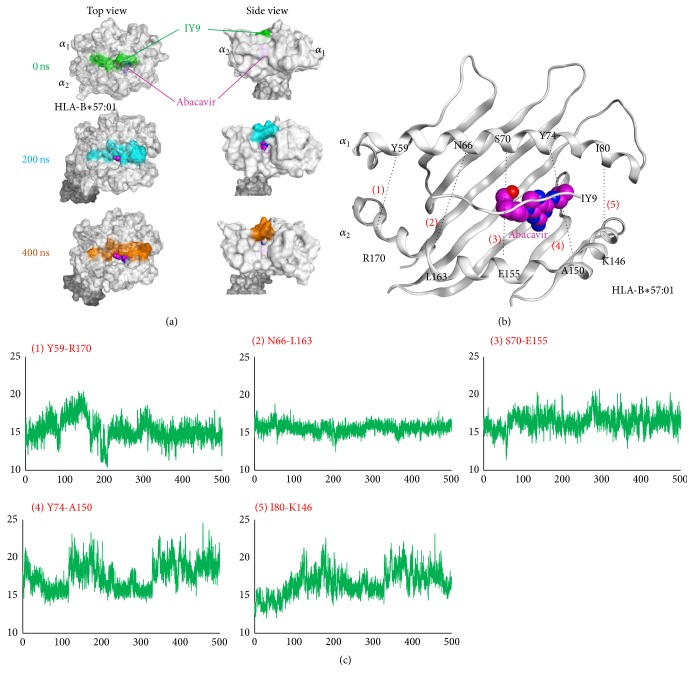
The distance between HLA-B*∗*57:01 *α*_1_ and *α*_2_ helices complexed with IY9 peptide became larger during MD simulation. (a) Top view (left) and side view (right) of IY9-HLA-B*∗*57:01 structures at 0, 200, and 400 ns. Abacavir was superposed with the structures of IY9-HLA-B*∗*57:01 complexes during the MD simulation. The IY9 peptide at 0, 200, and 400 ns is shown in green, cyan, and orange, respectively. HLA-B*∗*57:01 and the abacavir binding site are shown in gray and magenta, respectively. (b) Measurements of the distance between *α*_1_ and *α*_2_ helices of HLA-B*∗*57:01. IY9 peptide and HLA-B*∗*57:01 are shown in gray, and a superposed abacavir is shown in magenta. (c) The distance between *α*_1_ and *α*_2_ helices in the IY9-HLA-B*∗*57:01 complex. Horizontal and vertical axes of each graph represent time (ns) and RMSD values (Å), respectively.

To further confirm the partial detachment between self-peptides and HLA-B*∗*57:01, we visualized the structures of IY9-HLA-B*∗*57:01 complexes at 0, 200, and 400 ns (Figure 4(a)). These snapshots also revealed that the IY9 peptide was dissociated from the HLA-B*∗*57:01. Surprisingly, at 200 ns and 400 ns, the distance between the HLA-B*∗*57:01 *α*_1_ and *α*_2_ helices, which enable the peptides to be accommodated and presented, became larger. To confirm the more detailed characteristics, the five representative distances between *α*_1_ and *α*_2_ helices of the HLA-B*∗*57:01, (1) Y59-R170, (2) N66-L163, (3) S70-E155, (4) Y74-A150, and (5) I80-K146, were calculated (Figure 4(b)). As expected, the distances between (4) Y74-A150 and (5) I80-K146, which are both located near the abacavir binding site, became larger in comparison with their initial distances (17.1 Å (0 ns) and 19.2 Å (500 ns) for Y74-A150 and 12.7 Å (0 ns) and 17.5 Å (500 ns) for I80-K146) (Figure 4(c)). Together, these results suggested that increasing the distance between not only HLA and self-peptides but also HLA *α*_1_ and *α*_2_ helices is necessary for accommodation of abacavir in the peptide-binding cleft.

## 4. Discussion

Although DHRs are important problems that need to be resolved for both healthcare and pharmaceutical manufacturing, the mechanisms by which DHRs develop remain unclear. In this study, we performed MD simulations to validate a recently hypothesized mechanism (the altered-pHLA model) by which one of the most well-known DHRs, AHR, results in immediate activation of TCCs. For late hypersensitivity reactions of abacavir, Illing and her coworkers showed that abacavir binds specifically to HLA-B*∗*57:01, other than HLA-B*∗*57:03 or HLA-B*∗*58:01, using antigen-presenting cells [10]. However, to our knowledge, it is difficult to create* in vitro* assays to visualize a partial detachment of bounded peptide. Therefore, we carried out MD simulations in the current study. As it is well known, MD simulations are numerical representations of Newton's equations of motion [19]. This technique enables us to simulate the dynamics of peptides bound to HLAs, which is difficult to accomplish in* in vitro* or* in vivo* experiments. Increasing simulation times are being used for MD simulation studies [20, 21], and at least 10 ns to 400 ns MD simulation lengths are needed to monitor the dynamics of pHLA [22].

We performed 500 ns MD simulations before and after abacavir binding for the four pHLA complexes whose peptides were known to bind to HLA-B*∗*57:01. The calculated dynamics of these peptides with the RMSD values imply that certain peptides dissociated from the HLA. However, a previous study using other pHLA complexes showed that the peptide residues dissociated from the HLA-B*∗*27 with the RMSD values of up to 10.0 Å during 400 ns MD simulations [23]. Considering these results, our simulation results show much lower RMSD values (about 2 to 3 Å). Therefore, to confirm sufficient partial detachment between HLA and self-peptides for the drug to be accommodated, we measured the distances between the peptide and HLA residues near the abacavir binding site during MD simulations. For the IY9 peptide-HLA-B*∗*57:01 complex, the distances became larger up to 200 ns and then decreased gradually. The distances at 200 ns (15.7 Å (p7-Asp114), 13.3 Å (p7-Ser116), and 18.1 Å (p7-Ser116)) are large enough for the drug to be accommodated, because the minimum distances calculated from the crystal structures of abacavir-peptide-HLA complexes (PDB ID: 3UPR) are 7.6 Å, 9.3 Å, and 7.2 Å, respectively. After 200 ns, the distances decreased over time, especially for p7-Ser116 and p9-Ser116, suggesting that the peptide directs the attachment to the HLA.

The snapshots obtained from MD simulations of the IY9 peptide-HLA-B*∗*57:01 complex showed an enlarged distance not only between HLA and self-peptides but also between the *α*_1_ and *α*_2_ helices of HLA. The distances between HLA *α*_1_ and *α*_2_ helices, in particular those between residues Y74-A150 and I80-K146, became larger after 100 ns. However, the closure of these *α*_1_ and *α*_2_ helices was not confirmed during the period of our MD simulations. Performing MD simulations with a much longer timescale could be necessary for elucidating the complete mechanisms of the altered-pHLA model.

## 5. **C**onclusions

In this study, we performed MD simulations to elucidate the altered-pHLA model, which is a relatively new hypothesis to explain drug-induced immediate activation of TCCs, and concluded that one of our MD simulation results does indeed support this hypothesis. To our knowledge, this study is the first to test this hypothesis and support its validity. However, the results obtained from this study could not explain the detailed mechanisms by which only the IY9 peptide dissociates from HLA-B*∗*57:01. Future studies will be required to elucidate the detailed characteristics, such as the peptide specificity and the mechanisms by which this peptide dissociates from the HLA. Our findings might also be applied to other DHRs, such as flucloxacillin- and allopurinol-induced DHRs [24]. Such long-term MD simulations could contribute to the identification of DHRs and various other mechanisms about pHLA.

## Figures and Tables

**Table 1:** Self-peptides used for this study.

Peptides	Protein name (human)
LSSPVTKSF (LF9) (PDB ID: 3VH8)	Ig kappa chain C region
IAVKVNHSY (IY9)	E3 SUMO-protein ligase P1AS4
VAKVCQYTF (VF9)	NADH dehydrogenase [ubiquinone] 1 alpha subcomplex subunit 11
VTYKNVPNW (VW9)	GTF-binding nuclear protein Ran

Protein name represents the proteins bound to HLA before processing into the self-peptides [8]. LF9 is the peptide derived from the crystal structure and obtained from the Protein Data Bank (PDB) [14].
